# Ophthalmic Manifestations of Chlorine Gas Exposure: What Do We Know So Far?

**DOI:** 10.7759/cureus.35590

**Published:** 2023-02-28

**Authors:** Khayry Al-Shami, Salman Almurabi, Jafar Shatnawi, Khaled Qasagsah, Ghayda' Shatnawi, Abdulqadir J Nashwan

**Affiliations:** 1 General Practice, Jordan University Hospital, Amman, JOR; 2 Faculty of Medicine, Yarmouk University, Irbid, JOR; 3 Nursing, Hamad Medical Corporation, Doha, QAT

**Keywords:** eye disease, ocular manifestations, cornea, ophthalmology, chlorine gas

## Abstract

Chlorine gas is a hazardous substance that can cause severe health effects when inhaled or exposed to the skin. It is an odorless, colorless gas in many industrial and manufacturing settings and conflict areas. While exposure to chlorine gas is generally limited to the workplace and public areas, there are instances in which people may be exposed to high levels of chlorine gas for a short period of time due to spills, mishaps on the road or railroads, or other tragedies. In addition to the general health effects of chlorine gas, this essay will focus on the effects of chlorine gas on the eyes. The eyes are particularly sensitive to chlorine gas, and exposure can cause various symptoms, ranging from mild irritation to severe damage. Symptoms of chlorine gas exposure to the eyes include redness, burning, tearing, and blurred vision. In more serious cases, exposure to chlorine gas can cause permanent damage to the eyes, including corneal ulcers, scarring, and blindness. It is important to be aware of the signs and symptoms of chlorine gas exposure and the potential long-term effects to take the necessary steps to protect oneself. In addition to the potential health effects, it is important to understand the properties of chlorine gas. Chlorine gas is heavier than air and tends to settle in low-lying areas. It is highly reactive and can react with other substances to form hazardous compounds. As such, it is important to be aware of the potential for chlorine gas to react with other environmental substances and accumulate in certain areas. Finally, it is important to understand the background of chlorine gas use in various conflict areas. Chlorine gas has been used as a chemical weapon for centuries, and its use in modern warfare has been documented in various conflicts. As such, it is important to be aware of the potential for chlorine gas to be used in war zones and to take the necessary precautions to protect oneself. In conclusion, chlorine gas is a hazardous substance that can cause severe health effects when inhaled or exposed to the skin. The eyes are particularly sensitive to chlorine gas, and exposure can cause various symptoms, ranging from mild irritation to severe damage. It is important to be aware of the signs and symptoms of chlorine gas exposure and the potential long-term effects to take the necessary steps to protect oneself. Additionally, it is important to understand chlorine gas's properties and its background use in various conflict areas.

## Introduction and background

One of the chemicals produced and used the most in the modern world is chlorine. It is extensively used in several procedures, including those that treat water and bleach clothing and paper. Because of their intermediate water solubility and deeper penetration, chlorine-containing substances frequently cause immediate harm to bodily organs, including the eyes and respiratory system. The oxidation of functional groups in cell components, which results in the detrimental effects of the gas, is the mechanism of damage in chlorine exposure. Given that fish and other aquatic species are poisoned, it poses a major threat to marine life [1,2]. In normal conditions, chlorine is a yellowish-green gas with a density that is 2.5 times greater than that of air. Chlorine has a melting point of -100.98 degrees Celsius in a liquid state and a boiling point of -33.97 degrees Celsius. Chlorine is found in free form as a single molecule (Cl2) in the environment. Chlorine dissolves poorly in water but very well in various non-polar liquids. The majority of metals and a large number of non-metals interact with chlorine [3]. From time to time, we hear about the occurrence of chlorine gas leakage incidents, the most recent of which was the chlorine gas leakage in the port of Aqaba in Jordan on June 2022 due to the explosion of a tank containing 20 tons of a toxic chlorine gas, which led to 13 deaths and 300 people injured, with significant damage to the bodies of the people who were exposed to it, especially their eyes [4,5]. As we needed to understand more about chlorine gas, we concentrated on describing its symptoms in the eye, how to diagnose it, and how to deal with it.

## Review

History and epidemiology

Fritz Haber (born December 9, 1868, died January 29, 1934) was a German chemist and Nobel prize winner. He was also called the father of chemical warfare because he advised the German military to use chlorine gas as a chemical weapon [5]. Chlorine gas was first used as a chemical weapon on a large scale during World War 1 [6] when Germany was one of the first countries to use that gas [3]. Because of its devastation, it became prohibited by the Geneva protocol in 1925 [7]. Unfortunately, there were violations when it was used in the Iraq war in 2007 [8,9]. Hama is a governorate in Syria that was exposed twice to chlorine gas, and the first occurred on April 11, 2014 [10,11]. The second occurred on May 22, 2014 [12]; a case series study on 15 patients presenting to Kafr Zita hospital concluded many results; Table 1 shows the ophthalmic one [13]. And it has also been used from 2014 to 2017, when the Islamic State of Iraq and Syria (ISIS) used the chemical weapon (CW), mostly mustard and less often chlorine gases, against Peshmergas [14].

**Table 1:** Chlorine-exposed patient results by hospital admission status (n=15) *n=number

Demographics		Not hospitalized, n=7 (%)	Hospitalized, n=8 (%)	Total, n=15 (%)
Age (years)	Mean (range)	22.6 (SE 3.08)	28.5 (SE 7.02)	25.7 (2-59)
	under 15	1 (14)	2 (25)	3 (20)
	16-45	6 (86)	4 (50)	10 (67)
	46+	0 (0)	2 (25)	2 (13)
Sex	Female	3 (43)	4 (50)	7 (47)
	Male	4 (57)	4 (50)	8 (53)
Weight (kg)	Mean (range)	66.7 (SE2.07)	64.8 (SE10.9)	65.7 (15-100)
Ophthalmological	Itchy eyes	7 (100)	7 (88)	14 (93)
	Excessive lacrimation	7 (100)	8 (100)	15 (100)
Marital status	Unmarried	5 (71)	2 (25)	7 (47)
	Married	2 (29)	6 (75)	8 (53)
Pre-existing disease	None	7 (100)	5 (63)	12 (80)
	Arterial hypertension	0 (0)	1 (13)	1 (7)
	Obesity	0 (0)	2 (25)	2 (13)
Attack	11-Apr-14	0 (0)	7 (88)	7 (47)
	22-May-14	7 (100)	1 (13)	8 (53)

A plant in Gumi-Si, South Korea, accidentally released chlorine gas on March 5, 2013, and the incident was studied. They also utilized 209 non-hospitalized individuals' self-report questionnaires and the medical records of two patients admitted to the hospital. They discovered that eye discomfort was a symptom in 18.2% of the 209 non-hospitalized patients [15]. In another study of 10 patients who underwent simple spirometry following a high concentration of chlorine gas inhalation after a train derailment in Graniteville, South Carolina, there were nine males and one female with a mean age of 44.7 ± 16.1 years. Among the 10 patients, four were current smokers, five were nonsmokers, and one was a former smoker. They found that four of them complained of eye irritation [16].

A cross-sectional descriptive study was conducted on 163 male patients exposed to chemical weapons that contained mostly mustard and, less often, chlorine gas. When the ISIS used these weapons against Kurdish soldiers on the battlefield, by using separate interviews separately on a face-to-face basis for about 15-20 minutes, the most cited clinical manifestations were eye itching in 39.3% of the patients and eye redness in 34.4% of the patients. Table 2 describes these manifestations [14].

**Table 2:** Delayed ophthalmologic manifestations of Peshmergas exposed to chemical weapons No = number*

Eye manifestations	No.	%
Eye redness	56	34.4
Eye itching	64	39.3
Eye pain	17	10.4
Eye tearing	48	29.4
Photophobia	27	16.6
Foreign body sensation in the eye	7	4.3
Pterygium	7	4.3
Chronic conjunctivitis	22	13.5
Premature presbyopia	2	1.2

Physical properties and pathophysiology

Chlorine is a yellow-green, non-combustible gas with a pungent, irritating odor and is about two times heavier than air [8,17-18]. At temperatures below -30°F/-34°C or higher pressures of more than 778.8 kPa [18], chlorine is a clear to amber-colored liquid [17]. Chlorine deposits on hygroscopic surfaces, such as the eye, irritate it because of its partial solubility in water [15]. It was formerly thought that the dangerous effects of chlorine gas came from the free oxygen species that were created when it reacted with water. Cellular damage may, however, be caused by the oxidation of functional groups in cell components and the interaction of chlorine gas with tissue water, according to recent studies. However, this reaction can produce hypochlorous, hydrochloric acid, and free oxygen radicals, as shown in the equation below [19].

Cl2+ H2O <--> HCl + HOCl <--> 2HCl + O-

It is worth mentioning that this gas is reactive only locally; thus, systemic effects are not commonly observed. The acids formed from this reaction can react with the conjunctival mucous membrane and could rarely cause burns and corneal abrasions. The cause of its rarity is due to buffering by the tear film [8]. According to other studies, the epithelium lining's mucus and chlorine gas react to produce hydrochloric and hypochlorous acids. The oxidative byproduct hypochlorite plays a key role in modulating the effects of chlorine gas. Compared to chlorine gas, hydrochloric acid is 30 times less hazardous. Of course, the effects of chlorine gas are mostly due to its potent oxidative capabilities rather than its acidic composition [20].

Moreover, some mitochondrial dysfunction was seen. Reactive oxygen species (ROS) are produced by the mitochondria in a normal state, but because cells have complex systems for getting rid of them, they cannot harm the cell. Imagine, however, that the ROS increase following cell damage brought on by exposure to chlorine gas. As it will then be out of control, the ROS generated from damaged mitochondria may also harm healthy mitochondria, with both acute and delayed effects [21].

Clinical symptoms

Differences in the severity of symptoms vary from one another. The severity of symptoms depends on the concentration of gas exposure and duration of exposure [7]. Sixty tons of chlorine were released during the accident in Graniteville, South Carolina, USA, on January 6, 2005. Five hundred twenty-nine people looked for medical care, 311 were treated by local physicians, 220 had mild symptoms but did not look for medical treatment, eight died in the area accident, and one died in the hospital. Initial symptoms of chlorine gas exposure start to appear at a concentration of 1-3 ppm (parts per million), accompanied by an irritation of the mucus membranes. The eye irritation begins at 5-15 ppm alongside moderate upper respiratory tract irritation; 430 ppm leads to death in 30 minutes, and >1000 ppm results in death after a few minutes. The higher the concentration of chlorine gas, the worse the symptoms [22]. Eyes after exposure to chlorine manifest infections, abrasions, and corrosions in the conjunctiva that can be noticed [23]. In chlorine gas exposure, the first symptoms can be seen immediately or with a delayed onset after 24 hours of exposure [22].

Corneal injuries typically heal in one to two days and cause a burning sensation and superficial disruption of the corneal epithelium. Tearing, soreness, searing discomfort, conjunctival edema, conjunctivitis, lacrimation, blurred vision, a foreign body feeling, photophobia, corneal abrasions, and superficial punctate keratopathy have all been recorded as symptoms [24-28].

As one of the disinfectants used in swimming pools and tap water, chlorine damages the corneal epithelium. As a result, persons who frequent swimming pools frequently experience eye redness, itching, and irritation. Since chlorine is a disinfectant in swimming pools, an ophthalmic examination may reveal ciliary injection and superficial punctate keratitis [29].

Clinical diagnosis of chlorine gas exposure

The toxicity of chlorine gas is related to many factors, including the gas concentration, duration of exposure, and type of tissue where the moist tissues, such as lungs and eyes, are more likely to be affected. As we mentioned before, the harmful effect results either from chlorine gas itself or from its reaction with water in moist tissues that lead to forming byproducts, hypochlorous and hydrochloric acid, which then creates oxygen free radicals by other reactions [9,30-32]. Chlorine gas exposure accidents frequently occur, so we must diagnose exposed people based on exposure history, clinical presentation, biomarkers, and laboratory and radiological findings by a chemical, biological, radiological, and nuclear (CBRN)-trained physician [13,33].

Biomarkers

There are no recognized biomarkers for identifying victims of chlorine exposure or locating chlorine gas leaks or attacks. In addition, biomarkers aren't reliable for diagnosing patients exposed to chlorine gas due to the limited number still in pre-clinical experiments [34].

Laboratory Findings

Hypoxia: Metabolic acidosis may be due to hyperchloremia, which results from the absorption of hydrochloric acid following the reaction of chlorine gas with water, which could be confirmed by arterial blood gases (ABGs) [35,36].

Radiological

Chest X-ray (CXR) findings are often normal, but a CXR may be indicated to rule out pulmonary edema, pneumonitis, and acute respiratory distress syndrome (ARDS). In addition, we can use CT scans and endoscopy as diagnostic and management tools for patients with severe injury [37,38].

Eyes

Even though exposure to chlorine gas can cause corneal abrasions, where patients should be diagnosed if they have foreign body sensations, long-lasting pain, watery eyes, eye redness, blurred vision, hazy vision, and light sensitivity, eyes are rarely damaged severely [33,39].

**Figure 1:**
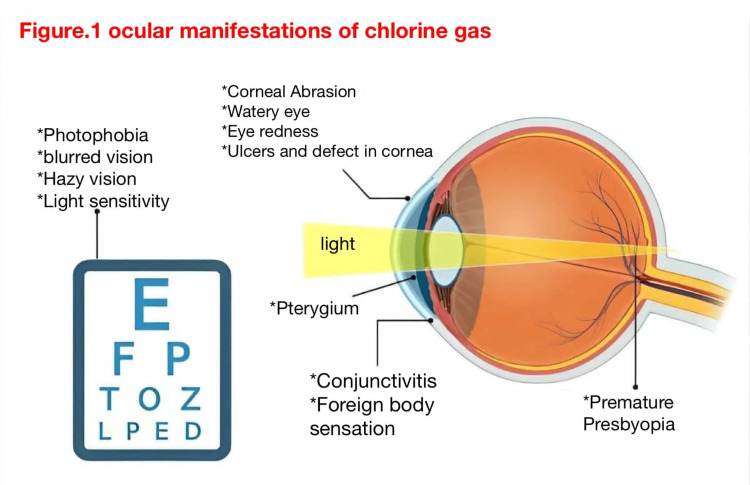
Ocular manifestations of chlorine gas The illustration was made by the authors.

To diagnose eyes, we can look for irritation, conjunctivitis, or corneal defects [40], in which chlorine gas reacts with the conjunctival mucous membranes [33]. Also, we can diagnose eyes by looking for the cornea from the central and peripheral points, which completely heal peripherally, but unfortunately, ulcer formation and scarring occur centrally (Figure 1) [33].

Detection of chlorine gas

Chlorine gas has a special odor, resembling a pepper and pineapple mixture. However, it has a strong odor and can be easily recognized [8,41] by humans at an approximate concentration of 0.2 ppm. Still, the irritant effect on the eyes and mucus membranes is 3-15 ppm (parts per million) [42]. It is important to remember that chlorine gas has a higher density than air, which causes it to be close to the ground and lengthen exposure times [33].

Treatment of chlorine gas exposure

Most of the time, treating chlorine gas exposure is supportive, and the first step in treating it is getting the people exposed to it away from the accident scene. As a result, emergency medical services must be fully prepared to save and assist patients in chlorine gas leak areas. Hence, victims should be sent immediately to the hospital from the chlorine gas leak area.

Ocular exposure to chlorine gas can be managed by irrigation of the eyes. An eye rinsing solution might be used for 3-5 minutes or using tap water for 15-20 minutes [43]. Contact lenses should be removed before flushing, a topical anesthetic is also recommended to make patients open their eyelids for better irrigation, and an ophthalmologist is required for any visible ocular injury [37].

After exposure to chlorine gas, patients can be categorized into three categories based on their symptoms: 1) patients with symptoms and major or minor injury, 2) patients with symptoms but no injury, and 3) patients without symptoms.

Patients without symptoms should be advised to avoid the area of the chlorine gas leak while patients with symptoms but without injury can take instructions for symptomatic treatment before discharge, Patients with major or minor eye symptoms, such as ocular pruritis, are treated with tap water wash of the eyes or take a steamy shower and eye drop preparations 10 OU (oculus uterque) q (quaque) I-2h. After receiving a topical anesthetic, it is recommended to rinse the eyes with one liter of saline in cases of severe conjunctivitis and corneal defects. If no corneal flaws exist, one drop of ophthalmic solution should be used OU for 1-2 hr [31].

The residual effects of warfare gases

According to the statistics of the war department, gassing caused the deaths of 70,752 troops. One thousand eight hundred forty-three (1,843) of the hospitalized casualties were gassed with chlorine [44]. The War Department analyzed 838 of these individuals and discovered that 96 required prolonged hospitalization, raising the potential that these soldiers may have residual effects from the gassing [45]. A total of 37 of the initial 838 patients were already deceased before this inquiry began. These fatal instances were also investigated to see if exposure to chlorine gas contributed to deaths several years after the Second World War. One of the postmortem findings from the ocular exam was acute conjunctivitis [46-49].

According to individual clinical studies, one of the instances evaluated following exposure to chlorine gas during a global war was a 34-year-old white guy who worked as a machinist before enlisting in the military. On October 14, 1918, he was badly "gassed" with chlorine; the initial symptoms included nausea, lethargy, and conjunctivitis. According to an examination, the lungs were healthy except for a few pleuritic rales near the base of the right axilla. The feet were swollen, and the eyes were swollen [48].

Another instance was investigated in which a 32-year-old white male worked as a clerk in a coal yard before enlisting in the military. On October 17, 1918, he received a little chlorine gas, followed by swollen eyes and purulent conjunctivitis. Based on the inspection, conjunctivitis was present on April 11, 1921. On January 23, 1923, previously undiagnosed "blurred eyesight" and chronic bronchitis were discovered during the follow-up checkup. The medical examiner reported that conjunctivitis was still present and that the patient had a dry, ineffective cough on July 30, 1926 [49].

## Conclusions

In a nutshell, chlorine is a non-combustible gas that is yellowish-green in color and nearly two times heavier than air. It has a strong, disagreeable odor. The quantity of chlorine gas, the length of exposure, and the kind of tissue all impact how toxic it is; wet tissues like the lungs and eyes are more susceptible to damage. Chlorine gas, or its reaction with water in wet tissues, which produces hypochlorous and hydrochloric acid as byproducts, has a negative effect. It is followed by other procedures that result in radicals lacking in oxygen. Many ocular symptoms, such as eye redness, irritation, tears, photophobia, chronic conjunctivitis, and many more, will be brought on by this. Due to the sensitivity and importance of the eye in daily life, we must be knowledgeable about this gas to handle any repercussions of chlorine exposure to the human body, whether it be a natural disaster or a war.
